# Patients’ Perspectives on Changes in Cancer Care During the COVID-19 Pandemic: Results of a Survey Among Patients with Gynecological Malignancies and Breast Cancer in Germany (NOGGO-S25/Expression XIII)

**DOI:** 10.3390/curroncol33080484

**Published:** 2026-08-17

**Authors:** Desislava Dimitrova, Jolijn Dirksje Boer, Dario Zocholl, Maja Krajewska, Hannah Woopen, Sara Nasser, Adak Pirmorady-Sehouli, Heike Jansen, Marion Kiechle, Jalid Sehouli

**Affiliations:** 1 Charité—Universitätsmedizin Berlin, Corporate Member of Freie Universität Berlin and Humboldt-Universität zu Berlin, Department of Gynecology with Center for Oncological Surgery, Augustenburger Platz 1, 13353 Berlin, Germany; 2 North-Eastern German Society for Gynecological Oncology (NOGGO), 13359 Berlin, Germany; 3 Charité—Universitätsmedizin Berlin, Corporate Member of Freie Universität Berlin and Humboldt-Universität zu Berlin, Institute of Biometry and Clinical Epidemiology, 10117 Berlin, Germany; 4 Charité—Universitätsmedizin Berlin, Corporate Member of Freie Universität Berlin and Humboldt-Universität zu Berlin, Department of Psychosomatic Medicine, 12203 Berlin, Germany; 5 Department of Obstetrics and Gynecology, TUM Klinikum Rechts der Isar, 81675 Munich, Germany

**Keywords:** COVID-19, ovarian cancer, breast cancer, gynecological cancer, cancer care, mental health, pandemic

## Abstract

Background/Objectives: The COVID-19 pandemic was a major challenge for healthcare systems and social life worldwide. During the pandemic, studies focused on examining structural changes in patient care and establishing new guidelines to manage COVID-19, but very few of them addressed patients’ perceptions and concerns regarding changes in cancer treatment during the pandemic. This study aimed to assess the changes in their therapy situation during three waves of the pandemic and the impact on their social life. Methods: This exploratory cross-sectional multicenter survey was conducted among patients with gynecological malignancies and breast cancer at gynecological oncology centers in Germany between October 2020 and February 2022. An anonymous paper-based 50-item questionnaire, available in German, assessed medical care, treatment modifications, mental health, and socioeconomic burden during the COVID-19 pandemic. Data were analyzed descriptively. Two subgroup analyses were conducted, comparing age groups ≤70 and >70 years, as well as patients with and without a migrant background. Results: Overall, 778 patients met the inclusion criteria of the survey (median age: 59; range: 24–93). In the majority of the patients (77.1%), the treatment schedules remained unchanged during COVID-19 pandemic. Treatment appointments were modified in a small number of patients (9.0%). Changes in therapy schedules were slightly more common among the second- and third-generation migrants and among patients ≤70 years. Despite the uncertainty of the COVID-19 pandemic, most patients (83.4%) kept their trust in the healthcare system. Acceptance of preventive measures was high, and 85.9% were willing to be vaccinated against COVID-19. Psychological symptoms such as worries and fear were reported by more than half of the participants during the last 2 weeks previous to the survey and more common among patients ≤70 years. Only 8.6% were more afraid of a COVID-19 infection than their cancer disease. Conclusions: Despite major challenges in cancer care due to the COVID-19 pandemic, access to cancer treatment and adequate management at gynecological oncology centers in Germany remained stable. The increased psychological burden in crises requires new infrastructure and should be addressed to improve patient care in future crises. The acceptance of taking preventive measures against COVID-19 was high among cancer patients.

## Simple Summary

During the COVID-19 pandemic, national lockdowns were implemented, restricting social life and personal contact, and healthcare systems worldwide were reorganized in order to treat an increased number of patients affected with SARS-CoV-2. Rearrangement affected cancer care due to the reallocation of medical staff and resources to COVID-19 care, staff illness and self-isolation, reduced operating theater capacity, and supply chain shortages. The present study aimed to assess how the COVID-19 pandemic affected therapy schedules in patients with gynecological malignancies and breast cancer in Germany and how the patients coped with the restrictions. Further, the survey explored whether there were differences among minority groups such as patients with migrant backgrounds and among older patients. The survey results showed that oncological treatment among different groups of patients remained largely unchanged, but the psychological burden increased.

## 1. Introduction

The outbreak of the COVID-19 disease, caused by severe acute respiratory syndrome coronavirus 2 (SARS-CoV-2) in January 2020 in Wuhan resulted in a pandemic in March 2020 [1]. It led to an unprecedented rearrangement of social life, travel, work, and the healthcare system worldwide. While the long-term effects of the pandemic are yet to be determined, its short-term implications resulted in changes in healthcare delivery, including the prioritization of emergencies and COVID-19 patients, a reduction in elective surgeries, and the postponement of scheduled appointments when feasible or their replacement with video-consultation [2,3].

Some of the first published studies reported that patients undergoing active cancer treatment were at a higher risk of severe COVID-19 outcomes, as well as higher postoperative morbidity and mortality [4,5]. In addition, the reorganization of healthcare services also affected cancer patients, who were not directly infected with COVID-19, leading to a decline in new cancer diagnoses in the first months of the pandemic [6]. Globally, one in five surgical patients with gynecologic cancer experienced modifications to their cancer management during the COVID-19 pandemic [7] and a delay in surgery between 2 and 8 weeks [7,8].

Some European studies were among the first to examine patients’ perspectives on changes in cancer care and showed that, despite significant challenges, patients trusted the healthcare system and were more concerned about disease progression and disruption of their treatment than about COVID-19 itself [9,10,11].

During the COVID-19 pandemic, increased levels of anxiety and depressive symptoms were reported across different cancer populations [12,13,14,15,16] and were associated with an increased likelihood of anxiety, depression, and poor well-being [12].

It is important to summarize the experiences and lessons learned from the COVID-19 pandemic in order to better understand the needs of patients in a crisis situation and develop better strategies to cope with challenges due to a disruption in standard medical care in case of future crisis situations. Some studies showed a disproportionate impact of COVID-19 on racial and ethnic minorities and disparities in outcome in vulnerable populations [17,18,19].

The primary goal of this survey was to assess whether an adequate level of cancer care was maintained during the COVID-19 pandemic among patients with gynecological and breast cancer, both with and without a migrant background, who were receiving active treatment or were in oncological follow-up and to explore what kind of changes were necessary to reorganize cancer care in gynecological oncology.

The secondary objective was to assess the psychological and socioeconomic burden on patients during the pandemic and how they coped with changes in their therapy schedule.

## 2. Materials and Methods

The anonymous multicenter cross-sectional patient survey “Expression XIII: Patients’ perspectives on changes in cancer care during the COVID-19 pandemic” was initiated by the Department of Gynecology with Center for Oncological Surgery at the Charité–Universitätsmedizin Berlin in collaboration with the North-Eastern German Society of Gynaecological Oncology (NOGGO). Between October 2020 and February 2022, patients were recruited during routine inpatient and outpatient visits at 12 gynecological oncology departments in Germany: two university hospitals in Berlin and Munich and 10 departments with secondary or maximal level of health care. Participation was voluntary and anonymous; informed consent was implied by the return of the complete questionnaire, and therefore, no written consent form was required.

All women ≥18 years with a history of primary or recurrent gynecological or breast cancer, who were treated in the departments and had willingness to participate during the study period, were eligible. The patients were directly approached by medical staff and asked whether they wished to participate. The presence of secondary malignancies (most common breast cancer) was assessed. Patients with a benign condition were excluded from the study.

The survey consisted of 50 questions, including single- and multiple-choice items, and was partially derived from instruments used in previous Expression surveys conducted by NOGGO [20,21,22,23]. Questions for assessing medical history, oncological treatment and health competence were adjusted from Expression II, III and VI Studies [20,21,23]. Questions for defining the migration background and satisfaction with medical care were adjusted from the Expression V Study [22]. Questions developed specifically for the for current survey addressed the medical care and lifestyle modifications related to the COVID-19 pandemic. Several questions assessing the fear of COVID-19 and changes in therapy schedules were adapted from the study of Gültekin and colleagues [9]. Mental health and concerns across various domains of daily life were also assessed using questions adapted from the German version of Distress Thermometer [24]. The questionnaire was available in the German language and administered in a paper-based format. The data were documented using REDCap version 16.0.40 (Research Electronic Data Capture tools hosted at Charité-University Medicine of Berlin) [25,26]. Before implementation, a pilot phase was conducted in a small patient cohort to evaluate the readability and comprehensibility.

The study was conducted in accordance with the Declaration of Helsinki and approved by the ethics committee of the Charité–Universitätsmedizin Berlin on 9 October 2020 (reference number of the research ethics committee: EA4/161/20).

Statistical analyses were carried out using R Statistical Software (v4.3.1; R Foundation for Statistical Computing, Vienna, Austria). Given the exploratory nature of the study, the main analyses were purely descriptive. Continuous variables are presented as medians with ranges, while categorical variables are reported as frequencies and percentages. All subgroup analyses were considered exploratory, and no prespecified hypotheses were tested. Consequently, *p*-values are reported as descriptive measures of the strength of evidence for potential group differences and should not be interpreted as confirmatory evidence. No adjustment for multiple comparisons was performed. Comparisons with *p*-values below 0.05 were considered to indicate potentially noteworthy differences deserving further investigation, using this threshold descriptively rather than as a criterion for confirmatory statistical significance. Categorical variables were compared across subgroups using Pearson’s Chi-squared test or Fisher’s exact test when the assumptions of the Chi-squared test were not met.

For continuous or ordinal variables, differences between two independent groups were assessed using the Wilcoxon rank-sum test, while comparisons across three or more independent groups were performed using the Kruskal–Wallis test.

For the statistical analysis, patients with ovarian, fallopian tube, peritoneal cancer were assessed together in the group of “ovarian cancer” and patients with endometrial, cervical, vaginal and vulvar cancer as “genital cancer”.

Two subgroup analyses were conducted: one comparing patients ≤70 years of age with those >70 years of age and another comparing patients without a migrant background to those who were first-, second-, or third-generation migrants. Due to the small number of patients, the second- and third-generation groups were combined for the analyses. The definition of migrant background and the classification in different generation was based on the criteria used in our previous Expression V Study [22].

## 3. Results

Between October 2020 and February 2022, 863 patients participated in the survey. Due to missing data about the main oncological diagnosis or medical conditions other than cancer, 85 were excluded from the analyses, and 778 patients with gynecological malignancies or/and breast cancer were eligible. The mean age at time of the survey was 59.6 (SD ± 13.2), the median age was 59.0 years (range: 24–93 years), and the majority of the patients had ovarian cancer (43.4%). The second most common malignancy was breast cancer (25.3%), and 7.5% had multiple cancer diseases (see Table 1).

More than half of the patients (*n* = 444; 58.0%) were married, and 555 (72.5%) had children. Around two-thirds (*n* = 507; 65.2%) shared a household with their spouse, 176 (22.6%) were living with their children, and 192 (24.7%) lived alone. Less than half 342 (44.5%) were employed, and 352 (46.1%) were retired. Most, 681 (88.7%), had access to the internet at home, and 740 (96.1%) owned a smartphone. The majority, 667 (87.1%), was born in Germany.

The majority of patients were undergoing active cancer treatment (*n* = 565; 73.6%), including surgery (*n* = 223; 28.7%), chemotherapy (*n* = 212; 27.2%), radiotherapy (*n* = 28; 3.6%), endocrine therapy (*n* = 54; 6.9%), and maintenance therapy (*n* = 103; 13.2%). Almost half of the patients, 328 (46.1%), stated that they were in cancer aftercare follow-up. The percentages sum to more than 100% because the categories for current treatment and follow-up care were not mutually exclusive (see Table 1).

Many patients reported comorbidities and regular medication use. The three most commonly reported conditions were 42.5% (*n* = 312) cardiovascular diseases, 24.6% (*n* = 169) polyneuropathy, and 22.9% (*n* = 160) thyroid disorders. The most commonly reported medications were antihypertensive drugs (45.1%), painkillers (33.9%), thyroid hormones (23.7%), and anticoagulants (18.7%).

### 3.1. Lifestyle and Prevention

Patients were asked about their levels of physical activity and changes in their health behaviors before and after the onset of the COVID-19 pandemic. The majority reported engaging in regular exercise: 28.8% (*n* = 220) exercised 1–2 h per week, 18.7% (*n* = 143) exercised 3–4 h per week, 16.3% (*n* = 125) exercised less than 1 h per week and 11.5% (*n* = 88) exercised 5 or more hours per week. A minority of patients (24.7%, *n* = 189) reported no regular exercise. Smoking was uncommon: 8.0% reported daily smoking, 4.4% reported occasional smoking, 50.0% never smoked, and 37.6% were former smokers. Only 0.5% of patients increased their tobacco consumption after the pandemic outbreak. Similar patterns were observed for alcohol consumption: one third (32.4%) of patients did not consume alcohol while 26.3% drank once per month, with 25.3% 2–4 times per month, 11.9% 2–3 times per week, and 4.1% four times per week. Only 2.2% reported an increase in alcohol consumption after the pandemic began. Dietary changes were reported by a small proportion of patients: 11.9% changed their diet, 7.6% began taking vitamins, and 3.5% reduced their carbohydrate intake.

Regarding vaccination attitudes, 642 patients (85.9%) were willing to receive a COVID-19 vaccination. Among the 105 patients (14.1%) who were skeptical about the vaccination, fear of side effects was the most commonly reported reason. Concerning other vaccinations, 56.7% had been vaccinated for influenza, 39.9% for pertussis, and 44.8% for pneumococcus.

### 3.2. Quality of Cancer Care 

Overall, patients were highly satisfied with the quality of cancer care during the pandemic, rating it on a 10-point scale (from 1—very dissatisfied to 10—very satisfied) with a mean of 7.8 and a median of 9. They were less satisfied with the restrictions on hospital visits for family and friends, giving a mean of 5.8 (median: 6). For most of the patients (63.8%), the availability of their doctors did not change, and 83.4% stated that their trust in the healthcare system was not affected by the pandemic. For 33.9% of patients, there was increased organizational burden in their treatment due to COVID-19-related measures.

### 3.3. Quality of Life 

**Figure 1:**
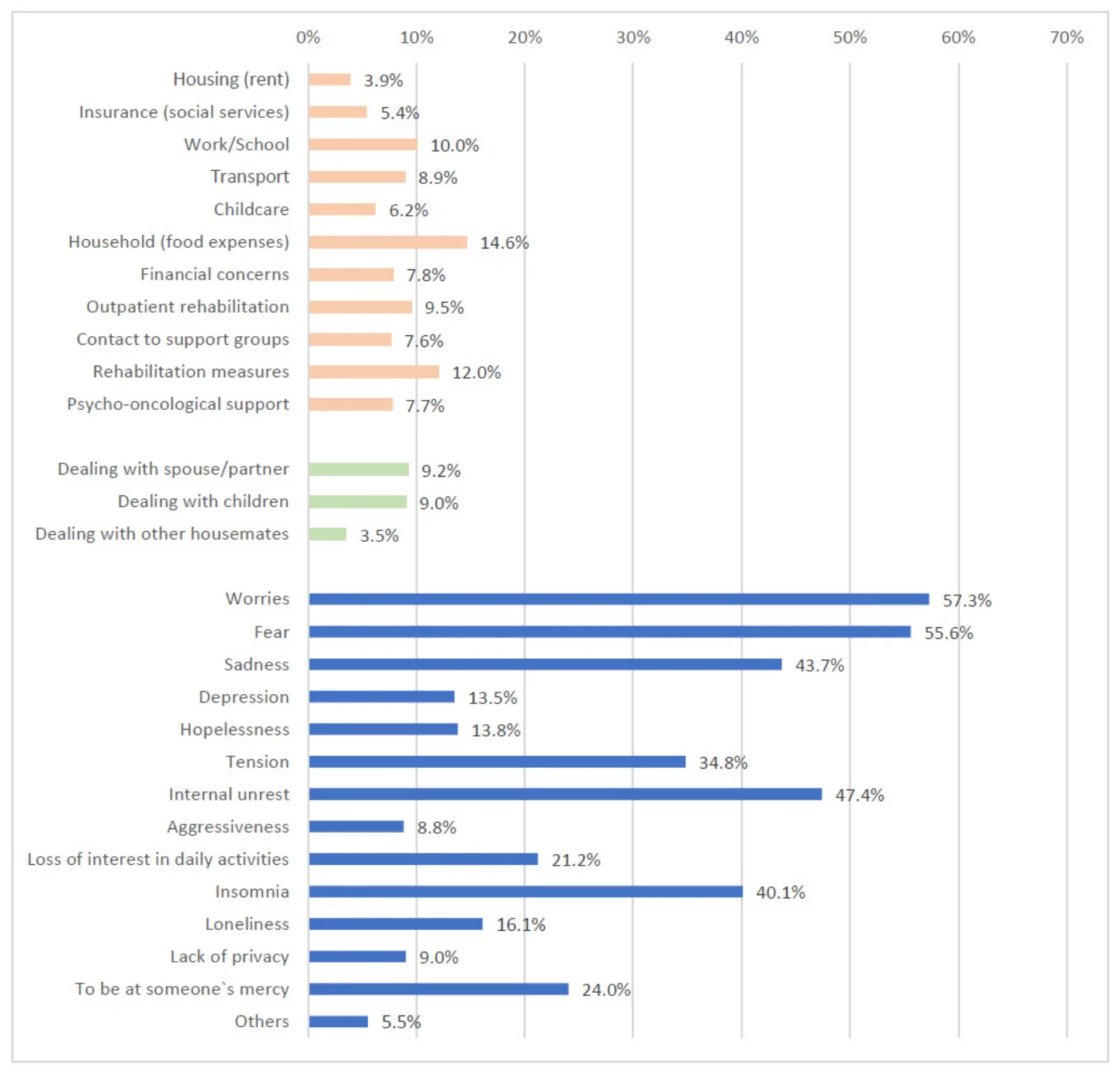
Psychological symptoms and daily-life worries (problems) in the last 2 weeks. Question: “Please indicate whether you have experienced any problems in any of the following areas in the last two weeks including today?”. Orange represents practical problems related to daily life and receiving healthcare support; green represents family problems; blue represents emotional and psychological problems.

Patients were asked about their mental health and daily life challenges during the two weeks preceding the survey. Worries (57.3%) and fear (55.6%) were reported by more than 50.0%. Additionally, 43.7% experienced sadness, 40.1% reported insomnia, and 47.4% experienced feelings of internal unrest. Regarding daily life problems, 14.6% experienced difficulties with household tasks, such as living costs, about 9.0% experienced challenges in managing relationships with children or spouses, and 10.0% had difficulties at work (see Figure 1).

The median distress, measured on a scale from 1 (maximum distress) to 10 (no distress at all), was 6 (range (Q1–Q3): 3.0–8.0) during the two-week period.

### 3.4. Changes in Cancer Care and Social Life

The majority of patients (68.9%) reported fearing their cancer more than COVID-19, and 33.4% had concerns that treatment delays due to the pandemic could result in cancer progression. Only 8.6% reported to have more fear of COVID-19 than cancer. More than half of the participants (58.7%) believed that cancer patients were at higher risk of contracting COVID-19 due to immune system changes, and 38.6% were afraid of getting the virus during hospital visits.

For most patients (*n* = 577; 77.1%), treatment schedules remained unchanged. Sixty-seven patients (9.0%) stated that their treatment had been adjusted, while 104 patients (13.9%) were unable to indicate whether any changes had occurred. The most common changes were a postponed appointment for surgery in 29 patients with a median of 8 days (5.5–17.0), a postponed CT/MRI appointment in 13 patients with a median of 10 days (2.5–28.0), a delay in chemotherapy in 8 patients with a median of 24.5 days (17.5–32.8), and a postponed follow-up appointment in 14 patients with a median of 35 days (28–60).

Around one-fifth of patients (19.8%) were enrolled in a clinical study. For only two patients (1.5%), participation was stopped due to the pandemic.

Among the participants, 197 (26.9%) reported a positive COVID-19 test in a family member or friend, and 45 (6.2%) reported exposure from medical staff at their treatment center. A total of 604 patients (82.3%) were tested for COVID-19, with 11 (1.9%) testing positive.

The most important source of support during the pandemic was family or friends (78.2%), followed by the attending physician (13.0%). The most trusted sources of information about COVID-19 were the treating physician (60.5%), family doctor (45.1%), information on television (27.9%), the internet (23.4%), printed booklets (21.5%), and social media (8.5%).

Telemedicine consultations were experienced by a small minority of the patients 6.2% (*n* = 45).

**Figure 2:**
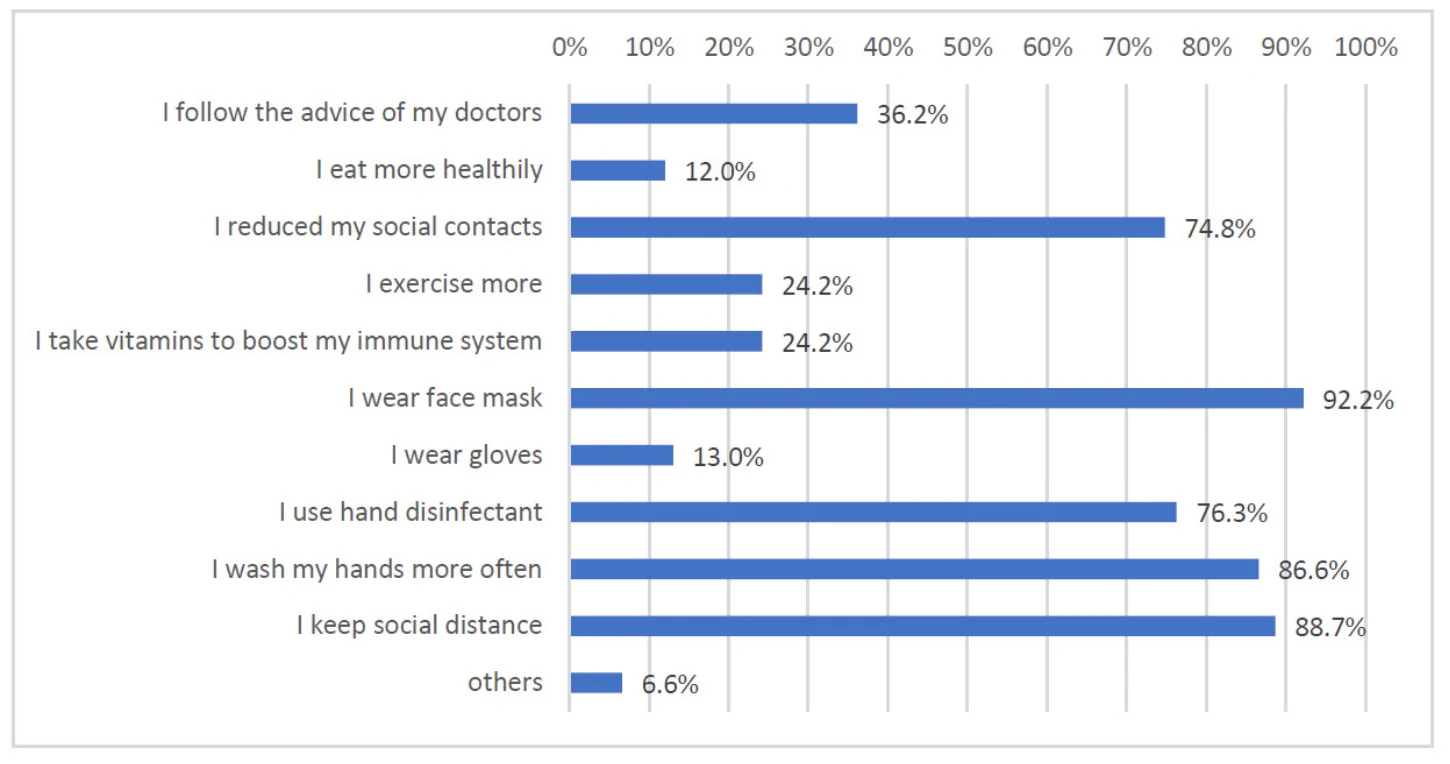
Preventive measures “What do you do to protect yourself against coronavirus infection?” (The question gave the possibility for multiple replies).

Adherence to preventive measures was high, with 92.2% wearing masks, 88.7% maintaining social distance, and 74.8% reducing social contacts (see Figure 2).

The most common coping strategies for social life restrictions during the pandemic included walking (59.1%), watching television or listening to music (59.3%), reading and writing (46.8%), using the internet including chatting or video calls (37.0%), manual activities such as knitting (17.9%), and yoga or meditation (17.5%).

The three most common suggested improvements in the current therapy situation during the COVID-19 pandemic from the patients’ perspective were a telephone COVID-19 hotline for questions (14.9%), 15% stated that psychological support will be helpful, and 10.9% requested a telemedicine consultation with the treating physician.

### 3.5. Subgroup Analyses

In the subgroup analysis, the majority of patients aged >70 years were retired, 54.7% shared a household with a spouse or partner, and 40.8% lived alone. One-third of patients older than 70 years (36.2%) had no internet access at home, and 11.2% did not own a smartphone. Older patients more frequently suffered from comorbidities compared to patients ≤ 70 years of age, such as cardiac disease (69.0% vs. 34.1%), stroke (4.0% vs. 1.3%), thromboembolism (22.3% vs. 12.0%), COPD (8.2% vs. 1.0%), asthma (17.4% vs. 8.7%), diabetes (15.1% vs. 4.2%), thyroid disorders (31.4% vs. 20.3%), renal dysfunction (11.5% vs. 5.9%), and polyneuropathy (32.7% vs. 22.2%). They were also more likely to be on regular medication compared with younger patients, which most probably represents an age-related effect.

Patients aged >70 years were less physically active but smoked less frequently compared to patients ≤70 years of age (3.9% vs. 15.3%), and 63.1% of them never smoked. They were more often vaccinated against influenza (79.5%) than patients aged ≤70 years (49.4%) and showed a higher willingness to receive a COVID-19 vaccination (94.2% vs. 83.5%).

Approximately one-third of patients younger than 70 years (30.3%) reported an increased psychological burden after the outbreak of the COVID-19 pandemic compared with 17.2% of older patients. Younger patients more frequently reported concerns about work or school (12.6%), childcare (7.5%), financial problems (9.5%), and partner relationships (10.5%). More patients aged ≤70 years in comparison to older ones experienced psychological symptoms such as worries (59.7%), fears (59.4%), hopelessness (15.7%), and sadness (47.2%).

Changes in therapy plans occurred more frequently in patients aged ≤70 years (9.4%) compared with those aged >70 years (5.9%) (*p* = 0.009) (see Table 2).

In the subgroup analyses according to migrant background, fewer differences were observed. The median age of first-generation migrants was 57 years, which was slightly younger than that of second- and third-generation migrants (median 58 years) and patients without a migration background (median 60 years). More than half of first-generation migrants (56.2%) held a university degree compared with 38.9% of second- and third-generation migrants and 35.3% of patients without a migrant background. Unemployment was most prevalent among first-generation patients, whereas women from the second and third generations exhibited the highest levels of employment.

Second- and third-generation migrants had a lower prevalence of cardiac disease (26.9%) compared with first-generation migrants (37.4%) and non-migrants (44.5%) (*p* = 0.014) and were less likely to take antihypertensive medication. When stratified according to age, the difference remained present only among the group of patients ≤70 years. This result may be related to the younger age of the migrants. Changes in therapy plans occurred slightly more often among second- and third-generation migrants (16.9%) than among first-generation migrants (14.3%) or patients without a migrant background (7.0%) (*p* = 0.022) (see Table 3).

## 4. Discussion

The aim of the present study was to evaluate whether significant changes in gynecological and breast cancer care occurred during the COVID-19 pandemic, to capture patients’ perspectives, and to explore how they coped with restrictions on daily life, work, and changes in their cancer treatment. Further, we aimed to explore whether certain groups such as older patients or migrants were more affected by changes in health care.

Within the scope of this study, we observed that cancer care delivery appeared to be maintained despite major challenges, with patients expressing continued trust in the healthcare system. Changes in therapy schedules were experienced slightly more often among second- and third-generation migrants and patients under 70 years of age, but the differences were minimal.

The study results underline that half of the patients suffered increased psychological burden, and younger patients had more worries regarding practical aspects of daily life, such as work, school, and childcare.

The cancer treatment remained unchanged for most patients in the current study. For only a few patients, there were changes, most commonly involving rescheduled physician appointments or planned CT/MRI examinations, as well as postponement of surgery, chemotherapy, or follow-up appointments. The percentage was higher for younger patients and second- and third-generation migrants. Similar results were reported by a European study during the first pandemic wave [9], where the majority of patients (64%) continued their care as planned, and only a minority (7.4%) did not attend treatment or follow-up appointments due to fear of COVID-19 infection. According to this study, appointments were cancelled by medical staff in 12.9% of cases, whereas postponement of planned treatment was a joint decision between patients and their treating team in 7.9% of cases. The data from GlobalSurg-CovidSurg Consortium showed that approximately 1 in 13 (7.9%) operations were cancelled and that 11.2% of patients experienced a significant delay (>8 weeks) to surgery, particularly those with ovarian cancer (15.7%) [7].

Similar changes were observed in a survey by a multicenter study [8], which found that the average waiting time for surgical appointments was prolonged by approximately two weeks compared with previous years (range 0–12 weeks). Additionally, surgical prioritization favored patients with early-stage disease (74%), primary disease presentation (61%), and good ECOG performance status (63%). Surgery had to be postponed in 29 patients of the 223 who were undergoing surgical therapy (13%) in the current study. Our survey was conducted during the second, third and fourth waves of the COVID-19 pandemic in Germany, and the percentages are higher but consistent with those reported by a study conducted during the same period (2020–2022) in the UK [27]. It observed a postponement of 5% (range 0–15%) of surgical cases during the second and third waves and 30% (range 16–57%) during the first wave in the UK. Our results are similar to a study from Germany, which compared two periods of time: first from 03/2019 to 02/2020 and second from 03/2020 to 02/2021, to explore the COVID-19 pandemic effect on elective general and visceral surgical procedures. It demonstrated a decrease of 14.8% for elective and 6.0% for emergency surgery procedures [28].

The majority of patients were satisfied with their cancer care. For most of them, the availability of their doctors did not change, and 83.4% stated that their trust in the healthcare system was not affected by the pandemic. The patients were less satisfied with restrictions imposed on hospital visits for family and friends. Their willingness to undergo self-protective measures was very high. These results are in line with the study of Recker et al., which took place before the present survey at the Department for Gynecology and Gynecological Oncology of the University Hospital Bonn (UKB) and the Department of Gynecology with Centre for Oncological Surgery Charité Berlin, and reported that the confidence in their physicians increased during the pandemic for 55.7% of the patients [10].

In our study cohort, for 33.9% of the patients, there was an increased organizational burden in their treatment due to COVID-19-related measures. The majority of patients (68.9%) reported fearing their cancer more than COVID-19, and 33.4% had concerns that treatment delays due to the pandemic could result in cancer progression. A study from the Netherlands [11], which investigated patients’ perspectives at the beginning of the first wave of the pandemic among 5302 cancer patients, reported treatment consequences in 30% of the cases, with chemotherapy and immunotherapy most frequently adjusted. Among patients in their study who experienced treatment delays or discontinuation, 55% and 63%, respectively, expressed concerns about the potential consequences of COVID-19-related changes for their cancer care. In the survey by Gültekin et al. 71% of patients expressed concern that delays or cancellations of their treatment or oncological follow-up could lead to cancer progression [9].

The impact of the COVID-19 pandemic on mental health in cancer patients was examined in the study by Lim et al. and was associated with increased likelihood of anxiety, depression, and poor well-being [12]. Another study by Nelson et al. among 2065 participants across the United States, Canada, and Europe in late March and early April 2020 demonstrated elevated anxiety and depressive symptoms compared to historical norms [16]. The findings were positively associated with COVID-19 concerns rather than with epidemiological factors.

More than half of the patients in the current survey reported experiencing anxiety and concerns during the last two weeks of the pandemic, and more patients ≤ 70 years of age reported psychological problems. Despite this increased psychological burden, only a small proportion reported increased tobacco or alcohol consumption. A review on changes in alcohol use during the COVID-19 pandemic found that 51% of individuals reported no change, 23% reported a reduction, and 26% reported an increase, with female gender, having children at home, higher educational level, and poorer mental health identified as factors associated with increased consumption [29]. Similar patterns were observed for tobacco use, with both increases and decreases reported [30].

Family and friends were the primary source of support for the majority of patients, followed by their treating physician. Physician consultations were also reported as the most important source of information, and most had experience with video consultations. However, some patients reported difficulties in accessing psycho-oncological support.

Strengths of the survey include that it was conducted in multiple centers and included a large sample of patients. Data were collected during the second, third and fourth waves of the COVID-19 pandemic, providing timely insights into patient experiences and perspectives. We performed subgroup analyses with focus on vulnerable groups.

Limitations include the use of a non-validated questionnaire and reliance on descriptive analyses of self-reported data; thus, some recall bias could not be excluded. The questionnaire was available only in German, excluding patients without sufficient language skills. Self-selection may have resulted in an overrepresentation of patients with stronger concerns. Due to patient recruitment at 12 gynecological oncology departments in Germany with a maximal level of health care, a certain center clustering effect could not be fully excluded and should be considered in the interpretation of the results. The survey did not include long-term follow-up, as psychological burden and behavioral changes were assessed at a single time point. Since participants were recruited during routine inpatient and outpatient visits, and the survey was administered during that same encounter, the total number of individuals approached was not documented. This approach was chosen to minimize disruption to routine clinical workflows and to avoid interfering with the delivery of patient care.

The study stretched over large period of time during the second, third and fourth COVID-19 waves in Germany, and there was no pre-pandemic comparison group or comparison between the different pandemic waves. The comparisons between the subgroups were mainly exploratory and not adjusted for confounders. Finally, due to cross-sectional design and explorative analyses, no causal relationship could be tested.

## 5. Conclusions

The results of the present study align with previous findings suggesting that, despite major challenges posed by the COVID-19 pandemic, cancer care for patients with gynecological malignancies and breast cancer appeared to be adequately maintained. Furthermore, our data indicate that patients’ trust in the healthcare system remained stable during this period. We identified an increased prevalence of mental health symptoms during the pandemic, which highlights a potential unmet need. From the patients’ perspectives, the use of telephone and video consultations, as well as additional psychological support, may represent valuable options to address this issue. Finally, these findings underscore the potential importance of structural changes, including the implementation of accessible psycho-oncological support, for future crises.

## Figures and Tables

**Table 1:** Patients’ characteristics.

	*N* (%)	N/A
Age (*N* = 763)		15
≤70 years of age	584 (76.5)	
>70 years of age	179 (23.5)	
Cancer entity (*N* = 778)		
Breast cancer	197 (25.3)	
Genital cancer	184 (23.7)	
Ovarian cancer	338 (43.4)	
Genital and breast cancer	7 (0.9)	
Ovarian and breast cancer	18 (2.3)	
Ovarian and genital cancer	29 (3.7)	
Ovarian, genital and breast cancer	5 (0.6)	
Migrant background (*N* = 760)		18
Without migrant background	592 (76.1)	
First generation	96 (12.3)	
Second generation	60 (7.7)	
Third generation	12 (1.5)	
Treatment status (*N* = 768)		10
Under current cancer therapy	Yes 565 (73.6) No 191(24.9) Not known 12 (1.6)	
Under follow-up care (*N* = 711)		67
	Yes 328 (46.1) No 383 (53.9)	
Therapy in a clinical study (*N* = 708)		70
	Yes 140 (19.8) No 421 (59.5) Not known 147 (20.8)	
Recurrence Status (*N* = 749)		29
Primary disease	252 (33.6)	
Recurrent disease	497 (66.4)	
Changes in therapy schedule due to COVID-19 pandemic (*N* = 748)		30
Yes	67 (9.0)	
No	577 (77.1)	
I do not know	104 (13.9)	

*N*: number; N/A: not available (missing values).

**Table 2:** Subgroup analysis based on age groups.

Patient Number ^#^	≤70 *N* = 584	>70 *N* = 179	*p*-Value
Age median (range), years	56 (48–62)	76 (73–80)	*p* < 0.001 *
Employment status			
Employed	338 (58.1)	0 (0.0)	*p* < 0.001 **
Unemployed	68 (11.7)	3 (1.7)	
Retired	176 (30.2)	176 (98.3)	
N/A	2	0	
Treatment and disease status			
Recurrent disease	196 (34.6)	49 (29.2)	*p* = 0.187 **
Under current cancer therapy	432 (74.9)	120 (68.2)	*p* = 0.175 ***
Chemotherapy	161 (27.6)	49 (27.4)	*p* = 0.959 **
Radiotherapy	23 (3.9)	4 (2.2)	*p* = 0.280 **
Operation	168 (28.8)	48 (26.8)	*p* = 0.612 **
Maintenance treatment	75 (12.8)	23 (12.8)	*p* = 0.998 **
Endocrine treatment	49 (8.4)	4 (2.2)	*p* = 0.004 **
Therapy in a clinical study	105 (19.5)	32 (20.1)	*p* = 0.974 **
Under follow-up care	259 (48.1)	61 (37.9)	*p* = 0.022 **
Changes in therapy schedule due to COVID-19 pandemic			
Yes	53 (9.4)	10 (5.9)	*p* = 0.009 **
No	421(74.8)	146 (85.9)	
I do not know	89 (15.8)	14 (8.2)	
N/A	21	9	
Psychological distress and contentment with therapy			
Distress score median (range Q1–Q3)	5.0 (3.0–8.0)	6.0 (4.0–8.0)	*p* = 0.205 *
Contentment with medical care median score (range Q1–Q3)	9.0 (7.0–10.0)	9.0 (6.0–10.0)	*p* = 0.393 *

* Wilcoxon rank test; ** Pearson’s Chi-squared test; *** Fisher’s Exact Test; N/A not available. # Only patients with recorded age are included in Table 2.

**Table 3:** Subgroup analysis based on migrant background.

	No Migrant Background	1st Generation	2nd and 3rd Generation	*p*-Value
Patient number	*N* (%) *N* = 592	*N* (%) *N* = 96	*N* (%) *N* = 72	
Age median (range), years	60 (52–70)	57 (47–68)	58 (48–66)	*p* = 0.021 *
Employment status				
Employed	255 (43.1)	43 (45.3)	41 (57.7)	*p* = 0.001 **
Unemployed	47 (7.9)	17 (17.9)	7 (9.9)	
Retired	290 (49.0)	35 (36.8)	23 (32.4)	
N/A	0	1	1	
Treatment and disease status				
Recurrent disease	189 (33.2)	30 (31.6)	25 (36.8)	*p* = 0.7806 **
Under current cancer therapy	435 (74.4)	66 (70.2)	53 (73.6)	*p* = 0.879 ***
Chemotherapy	153 (25.8)	33 (34.4)	22 (30.6)	*p* = 0.179 **
Radiotherapy	21 (3.5)	3 (3.1)	3 (4.2)	*p* = 0.938 **
Operation	180 (30.4)	21 (21.9)	20 (27.8)	*p* = 0.225 **
Maintenance treatment	84 (14.2)	8 (8.3)	8 (11.1)	*p* = 0.250 **
Endocrine treatment	42 (7.1)	6 (6.2)	5 (6.9)	*p* = 0.955 **
Therapy in a clinical study	98 (17.9)	24 (30.4)	14 (20.6)	*p* = 0.015 **
Under follow-up care	250 (46.1)	39 (44.8)	29 (43.9)	*p* = 0.928 **
Changes in therapy schedule due to COVID-19 pandemic				
Yes	40 (7.0)	13 (14.3)	12 (16.9)	*p* = 0.022 **
No	447 (78.6)	67 (73.6)	50 (70.4)	
I do not know	82 (14.4)	11 (12.1)	9 (12.7)	
N/A	23	5	1	
Psychological distress and contentment with therapy				
Distress score median range (Q1–Q3)	5.0 (3.0–8.0)	7.0 (3.5–8.0)	5.0 (3.0–8.0)	*p* = 0.245 *
Contentment with medical care score median range (Q1–Q3)	9.0 (7.0–10.0)	9.0 (6.5–10.0)	8.0 (5.0–10.0)	*p* = 0.303 *

* Kruskal-Wallis test, ** Pearson’s Chi-squared test; *** Fisher’s Exact Test, N/A not available.

## Data Availability

The data that support the findings of this study are not publicly available due to privacy and ethical restrictions.

## Abbreviations

The following abbreviations are used in this manuscript:

COVID-19	Coronavirus disease 2019
SARS-CoV-2	Severe acute respiratory syndrome coronavirus 2
FIGO	International Federation of Gynaecology and Obstetrics
ECOG	Eastern Cooperative Oncology Group Performance Status.
CT	Computed tomography
MRI	Magnetic resonance imaging
REDCap	Research Electronic Data Capture
NOGGO	North-East German Society for Gynecological Oncology
UKB	University Hospital Bonn
